# AmphiBIO, a global database for amphibian ecological traits

**DOI:** 10.1038/sdata.2017.123

**Published:** 2017-09-05

**Authors:** Brunno Freire Oliveira, Vinícius Avelar São-Pedro, Georgina Santos-Barrera, Caterina Penone, Gabriel C. Costa

**Affiliations:** 1 Departamento de Ecologia, Universidade Federal do Rio Grande do Norte, Lagoa Nova, Natal, RN 59072-970, Brazil; 2 Department of Wildlife Ecology and Conservation, University of Florida, Gainesville, FL 32611-0430, USA; 3 Departamento de Biología Evolutiva, Facultad de Ciencias, Universidad Nacional Autónoma de México, Circuito Exterior S/N, Ciudad Universitaria 04510, México; 4 Department of Biology, Auburn University at Montgomery, Montgomery, AL 36117, USA

**Keywords:** Macroecology, Data integration, Herpetology

## Abstract

Current ecological and evolutionary research are increasingly moving from species- to trait-based approaches because traits provide a stronger link to organism’s function and fitness. Trait databases covering a large number of species are becoming available, but such data remains scarce for certain groups. Amphibians are among the most diverse vertebrate groups on Earth, and constitute an abundant component of major terrestrial and freshwater ecosystems. They are also facing rapid population declines worldwide, which is likely to affect trait composition in local communities, thereby impacting ecosystem processes and services. In this context, we introduce AmphiBIO, a comprehensive database of natural history traits for amphibians worldwide. The database releases information on 17 traits related to ecology, morphology and reproduction features of amphibians. We compiled data from more than 1,500 literature sources, and for more than 6,500 species of all orders (Anura, Caudata and Gymnophiona), 61 families and 531 genera. This database has the potential to allow unprecedented large-scale analyses in ecology, evolution, and conservation of amphibians.

## Background & Summary

Organisms’ life forms and ecological strategies (simply referred to as ‘traits’) reflect the outcome of continuous evolutionary pressures by biotic and abiotic factors^1,2^. Traits strongly determine the species’ ability to persist in a variety of environments, including interactions with other species^2–5^. At evolutionary time scales, the expression of new traits may create opportunities for phylogenetic lineages to explore novel niches, escape from predation or competition, and hence promote speciation by adaptive radiation^6–8^. At the ecological scale, traits are especially relevant in the study of community assembly where species coexistence is determined by different processes that influence trait composition of the community (e.g., coexisting species share more or less similar traits than expected by chance)^4,9,10^. Furthermore, species traits are linked to ecosystem functions and services necessary for human well-being (e.g., burrowing behavior alters soil properties, body size is associated with animal nutrient transport capacity, and feeding habits control food web structure)^11–14^. However, recent biodiversity loss due to anthropogenic causes raise questions about the ability of ecosystems to continue providing these benefits^15^. Therefore, understanding the mechanisms influencing patterns in trait diversity (or functional diversity), including human disturbance, is increasingly needed in face of rapid global changes^16,17^.

The last decade experienced a surge in the availability of natural history trait (i.e., morphological, ecological and reproduction traits) databases with broad taxonomic coverage^18–22^ allowing unprecedented broad scale approaches in ecology and evolution^23–26^. Such data are still scarce for many amphibian species^27–29^. Amphibians are among the most diverse vertebrate groups on Earth, with more than 7,400 species and dozens of new species described every year^30^. They are abundant in many terrestrial and freshwater ecosystems, where they perform important ecosystem functions^31,32^. They are also the most threatened vertebrate group worldwide, with many species on the edge of extinction^33,34^. As such, it is urgent to improve our knowledge on amphibian traits in order to assess and predict their response to environmental changes and create conservation strategies that guarantee their survival.

In this context, we introduce AmphiBIO, an extensive database containing natural history traits for 6,775 amphibian species globally. AmphiBIO releases information on error-checked and referenced traits related to ecology, morphology and reproduction features of amphibians. Trait information was assembled from more than 1,500 literature sources, including peer-reviewed papers, existing life history databases, and other aggregated sources, in order to stimulate more comprehensive research in ecology, evolution, and conservation of amphibians. To enhance data quality, we implemented a protocol in which incorporated data were double-checked for potential errors. By making this data available to the scientific community we aim to advance the sharing of biological data and support a more integrative trait-based evolutionary and ecological science.

## Methods

In order to select a comprehensive set of relevant traits, while being as efficient as possible on the balance between searching effort and data collection, we surveyed amphibian traits commonly reported in scientific literature and/or existent in smaller data sets. Based on this initial survey, we selected 17 traits related to amphibian ecology, morphology, and reproduction, as described in Table 1. These traits reflect a variety of ecological strategies, niches, and functional roles, also collected for other vertebrate groups, which opens possibilities of joint analyses with other databases (e.g., PanTHERIA^18^, EltonTraits^20^, and^21^).

We conducted a systematic search in the literature primarily from peer-reviewed scientific publications, accessed on-line through academic platforms such as Google Scholar, Web of Science and Zoological Record, although printed material was also consulted whenever available. Furthermore, we assembled data from books, field guides, specialized websites (e.g., amphibiaweb.org), and from gray literature (e.g., technical reports, government documents, monographs, theses and dissertations). We also incorporated data from eleven smaller pre-existing data sets available on-line or kindly provided by their authors (Table 2), and previous data compilations by the authors.

We followed Frost^30^ for taxonomy. Due to the increasing number of amphibian species discovered every year, we limited our taxonomic coverage on the list of species described until 2011 (6,775 species)^30^. We standardized taxonomic classification from the various literature sources, as well as resolved formatting inconsistencies or spelling errors, using the Amphibian Species of the World website (http://research.amnh.org/vz/herpetology/amphibia/). Future versions of AmphiBIO will include newly discovered species and taxonomic revisions following Amphibian Species of the World.

## Data Records

AmphiBIO can be downloaded from *Figshare* repository record (Data Citation 1) under a CC-BY license, which permits distribution of derivatives.

**Figure 1:**
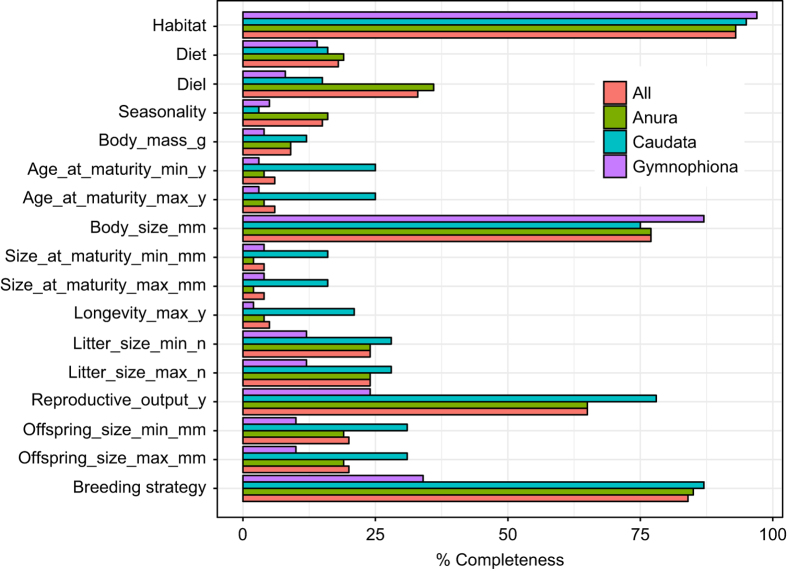
Percentage of data completeness for each trait released in AmphiBIO v1. Values are presented by Amphibian Order (Anura, Caudata and Gymnophiona) and for all combined Amphibian species. In order to better depict data completeness, the different categories of Habitat (Fos, Ter, Aqua, Arb), Diet (Leaves, Flowers, Seeds, Fruits, Antro, Vert), Diel (Diu, Noc, Crepu), Seasonality (Wet_warm, Wet_cold, Dry_warm, Dry_cold) and Breeding strategy (Dir, Lar, Viv), are presented together. Variable descriptions are in Table 1.

Details for the trait data are summarized in Table 1. In total, we extracted and aggregated the data from the 1,788 literature sources. The data provided here has 30% of data completeness, revealing the limited knowledge on life history traits for most amphibians^31,35,36^. Analyzing by Order, this corresponds to 29.5% of possible data for Anura, 35.4% for Caudata and 20% for Gymnophiona (Fig. 1). The lower average data completeness for Gymnophiona is probably related to fossorial and cryptic habits that make ecological data scarcer for this group^35^. In addition, missing information for Gymnophiona is in accordance with the vast percentage of data deficient species (e.g., on the IUCN redlist). However, data completeness varies largely among traits (Fig. 1). Specifically, traits representing overall habitat use, body size and coarse reproduction features (Reproductive_output_y and Breeding strategy) received the larger proportion of data completeness. In contrast, traits related to Diet, time of activity (Diel and Seasonality), body mass, age at maturity, size at maturity, longevity, litter size and offspring size, were more difficult to obtain, and missing information was more common.

For categorical traits, such as Habitat, Diet, Diel and Seasonality, we reported multiple trait categories if documented so in the literature (Table 1). For instance, a frog may be documented by one author as ground-dwelling, but may have been found perching on trees by other authors. In this case, we reported both terrestrial and arboreal behaviors for that species. We adopted a binary classification for categorical traits, where a 1 was assigned if a specific trait category was recorded in the literature for a given taxon, and a NA was assigned if a specific trait category has never been recorded in the literature for a given taxon. We advise caution when interpreting NA, considering that a given trait may not be definitely absent, but rather it has never been reported so in the literature, at least to our knowledge. By adopting this categorization scheme, we hope to accommodate the lack of sufficient ecological knowledge for the vast majority on amphibian species^31,35,36^. We do not use relative importance of trait categories as this information was absent in most literature searches; therefore we assume trait categories as equally important.

In Anura, we reported body size as snout to vent length (SVL). In Gymnophiona and Caudata, body size is reported as total length (TL). When TL was not reported for Caudata, but individual measurements for SVL and tail length were available, we reported TL as the sum of SVL and tail length. Given tail autotomy in several groups, some references report only SVL for Caudata. In these cases, we reported SVL and flagged this information in the field ‘OBS’. There is no standardization for the measurement of egg size in amphibians. Sometimes this measure is presented in the literature as ‘vitellus diameter’, which considers embryo dimensions. It can also be referred to as ‘total diameter’, when the thickness of external jelly capsules is also considered. As a rule, our data on egg size refer to vitellus diameter, except for the species of which there are only available data for total diameter. However, this later information is not present in the database. Further details for each trait data is given in Table 1. Moreover, any issue we considered important at the moment of data searching, such as discordances between current species name and the name in the literature record, was reported in the field ‘OBS’.

## Technical Validation

We implemented three procedures to detect inconsistency in data entry before publishing the first version of this database. Data identified as outliers by any of the adopted validation procedures were flagged, checked for validity based on multiple literature sources, and either corrected or purged whenever necessary from the database.

Firstly, we used Bonferroni’s test of Studentized residuals to identify outliers in continuous variables that were unusual with respected to allometric relationships with body size^37^. Because there may be vast differences in trait variation between different taxonomic levels, in order to maximize the detection of outliers that could be checked for validity we repeated this procedure considering allometric relationships within taxonomic levels (i.e., Order, Family and Genus). To have sufficient statistical power, we omitted clades composed by less than 6 species with data. Secondly, we used ANOVA with taxonomic level as the grouping variable to identify data values that were significant outliers from general trends (standardized residual >3)^38^. Similarly to Bonferroni’s test, we fitted separate one-way ANOVAs for each taxonomic level (i.e., Order, Family and Genus), and omitted clades composed by less than 6 species. Thirdly, we applied the Attribute Value Frequency (AVF) algorithm to detect outliers in categorical variables^39^. AVF score represents the infrequentness of an attribute value by calculating the number of times this value is found in the dataset^39^. In all cases, correction was applied whenever necessary.

A total of 11,614 cells from our database were flagged and validated. We found an error rate of ~0.01% and no other errors after another 100 random data sample. AmphiBIO will undoubtedly benefit from further quality control and curation. We aim to facilitate this process by sharing through this archive online (see Usage Notes).

## Usage Notes

The data release is available from *Figshare* repository record (Data Citation 1) in a compressed (.zip) folder containing four files as described below. Lack of information is indicated as NA.

### Identity:

(1) AmphiBIO_v1.csv

(2) AmphiBIO_v1_references.csv

(3) AmphiBIO_v1_literature_cited.pdf

(4) Metadata.pdf

### Format:

(1) ASC file, Windows-1,252 encoding.

(2) ASC file, Windows-1,252 encoding.

(3) PDF file.

(4) PDF file.

### Header information (Headers describe the information of each column):

(1) id, Order, Family, Genus, Species, Fos, Ter, Aqu, Arb, Leaves, Flowers, Seeds, Fruits, Arthro, Vert, Diu, Noc, Crepu, Wet_warm, Wet_cold, Dry_warm, Dry_cold, Body_mass_g, Age_at_maturity_min_y, Age_at_maturity_max_y, Body_size_mm, Size_at_maturity_min_mm, Size_at_maturity_max_mm, Longevity_max_y, Litter_size_min_n, Litter_size_max_n, Reproductive_output_y, Offspring_size_min_mm, Offspring_size_max_mm, Dir, Lar, Viv, OBS.

(2) id, Order, Family, Genus, Species, Reference.

(3) Does not apply.

(4) Does not apply.

## Additional Information

**How to cite this article:** Oliveira, B. F. *et al.* AmphiBIO, a global database for amphibian ecological traits. *Sci. Data*. 4:170123 doi: 10.1038/sdata.2017.123 (2017).

**Publisher’s note:** Springer Nature remains neutral with regard to jurisdictional claims in published maps and institutional affiliations.

## Supplementary Material



## Figures and Tables

**Table 1:** Overview of variables included in the AmphiBIO database.

**Variable name**	**Variable definition**	**Unit**
**id**	AmphiBIO species’ identification number.	N/A
**Order**	Amphibian Species of the World Order (Frost^30^).	N/A
**Family**	Amphibian Species of the World Family (Frost^30^).	N/A
**Species**	Amphibian Species of the World species scientific name (Frost^30^).	N/A
**Habitat**	Overall vertical foraging stratum classification. Ignores details about seasonal or ontogenetic changes.	
**Fos**	Fossorial.	Binary
**Ter**	Terrestrial.	Binary
**Aqua**	Aquatic.	Binary
**Arb**	Arboreal.	Binary
**Diet**	Food items from the eating habits of adults using qualitative dietary categories. Information is based of specialist guess, direct observation or stomach content examination, as reported in the literature.	
**Leaves**	Species eat leaves.	Binary
**Flowers**	Species eat flowers.	Binary
**Seeds**	Species eat seeds.	Binary
**Fruits**	Species eat fruits.	Binary
**Arthro**	Species eat arthropods.	Binary
**Vert**	Species eat vertebrates (includes cannibalism).	Binary
**Diel**	Overall diel period as active.	
**Diu**	Diurnal (i.e., active during the day).	Binary
**Noc**	Nocturnal (i.e., active during the night).	Binary
**Crepu**	Crepuscular (i.e., active during the period immediately after dawn and that immediately before dusk).	Binary
**Seasonality**	Seasonal period as active. Based on the comparison of the precipitation (wet or dry) and temperature (warm or cold) conditions when active in relation to the average climatic conditions over the year. Climatic conditions were obtained from weather stations closer to localities where specimens were collected or to field sites reported in publications (available at www.weatherbase.com).	
**Wet_warm**	Active is during wet and warm months.	Binary
**Wet_cold**	Active is during wet and cold months.	Binary
**Dry_warm**	Active is during dry and warm months.	Binary
**Dry_cold**	Active is during dry and cold months.	Binary
**Body_mass_g**	Maximum adult body mass.	Grams
**Age_at_mature_min_y**	Minimum age at maturation/sexual maturity.	Years
**Age_at_mature_max_y**	Maximum age at maturation/sexual maturity.	Years
**Body_size_mm**	Maximum adult body size. In Anura, body size is reported as snout to vent length (SVL). In Gymnophiona and Caudata, body size is reported as total length (TL).	Millimeter
**Size_at_mature_min_mm**	Minimum size at maturation/sexual maturity.	Millimeter
**Size_at_mature_max_mm**	Maximum size at maturation/sexual maturity.	Millimeter
**Longevity_max_y**	Maximum life span.	Years
**Litter_size_min_n**	Minimum no. of offspring or eggs per clutch.	Number
**Litter_size_max_n**	Maximum no. of offspring or eggs per clutch.	Number
**Reproductive_output_y**	Maximum no. reproduction events per year.	Number
**Offspring_size_min_mm**	Minimum offspring or egg size.	Millimeter
**Offspring_size_max_mm**	Maximum offspring or egg size.	Millimeter
**Breeding strategy**	Whether the species reproduce via direct, larval development or is viviparous.	
**Dir**	Species reproduce via direct development.	Binary
**Lar**	Species present larval stages.	Binary
**Viv**	Species is viviparous.	Binary
**OBS**	Misc. comments.	N/A

**Table 2:** Data sets incorporated to the AmphiBIO database.

**Incorporated data**	**Reference**
Body mass, clutch size and egg size for 114 species of Australia species.	^40^
Habitat for 5,717 species of amphibians.	^41^
Body size of 455 species of species lists for regional assemblages throughout the World.	^42^
Body size for 1,825 species of amphibians.	^43^
Body mass, clutch size, age at maturity and longevity for 54 species of Dendrobatidae.	^44^
Body mass, age at maturity and longevity for 33 species of Urodela and 86 Anura.	^45^
Body size for 534 species and egg size and clutch size for 119 species of Anura.	^46^
Habitat and annual reproductive output for amphibians.	^47^
Body size and clutch size for 718 Anuran species.	^48^
Body size, body mass, clutch size and age at maturity for 86 Anura and Urodela from Europe.	^28^
Body size for 356 Anura and Urodela from North America and Europe.	^49^
